# Increased CD4^+^ T cell lineage commitment determined by CpG methylation correlates with better prognosis in urinary bladder cancer patients

**DOI:** 10.1186/s13148-018-0536-6

**Published:** 2018-08-03

**Authors:** Emma Ahlén Bergman, Ciputra Adijaya Hartana, Markus Johansson, Ludvig B. Linton, Sofia Berglund, Martin Hyllienmark, Christian Lundgren, Benny Holmström, Karin Palmqvist, Johan Hansson, Farhood Alamdari, Ylva Huge, Firas Aljabery, Katrine Riklund, Malin E. Winerdal, David Krantz, A. Ali Zirakzadeh, Per Marits, Louise K. Sjöholm, Amir Sherif, Ola Winqvist

**Affiliations:** 1 Unit of Immunology and Allergy, Department of Medicine Solna, Karolinska Institutet, Karolinska University Hospital, Stockholm, Sweden; 2 Department of Urology, Sundsvall Hospital, Sundsvall, Sweden; 3 Department of surgical and perioperative Sciences, Urology and Andrology, Umeå University, Umeå, Sweden; 4 TLA Targeted Immunotherapies AB, Stockholm, Sweden; 5 Department of Urology, Akademiska University Hospital, Uppsala, Sweden; 6 Department of Surgery, Urology Section, Östersund County Hospital, Östersund, Sweden; 7 Centre for Research and Development, Faculty of Medicine, Uppsala University, County Council of Gävleborg, Uppsala, Sweden; 8 Department of Urology, Västmanland Hospital, Västerås, Sweden; 9 Department of Clinical and Experimental Medicine, Division of Urology, Linköping University, Linköping, Sweden; 10 Department of Radiation Sciences, Diagnostic Radiology, Umeå University, Umeå, Sweden; 11 Center for Molecular Medicine, Department of Clinical Neuroscience, Karolinska Institutet, Stockholm, Sweden

**Keywords:** DNA methylation, CD4-positive T lymphocytes, Urinary bladder neoplasms

## Abstract

**Background:**

Urinary bladder cancer is a common malignancy worldwide. Environmental factors and chronic inflammation are correlated with the disease risk. Diagnosis is performed by transurethral resection of the bladder, and patients with muscle invasive disease preferably proceed to radical cystectomy, with or without neoadjuvant chemotherapy. The anti-tumour immune responses, known to be initiated in the tumour and draining lymph nodes, may play a major role in future treatment strategies. Thus, increasing the knowledge of tumour-associated immunological processes is important. Activated CD4^+^ T cells differentiate into four main separate lineages: Th1, Th2, Th17 and Treg, and they are recognized by their effector molecules IFN-γ, IL-13, IL-17A, and the transcription factor Foxp3, respectively. We have previously demonstrated signature CpG sites predictive for lineage commitment of these four major CD4^+^ T cell lineages. Here, we investigate the lineage commitment specifically in tumour, lymph nodes and blood and relate them to the disease stage and response to neoadjuvant chemotherapy.

**Results:**

Blood, tumour and regional lymph nodes were obtained from patients at time of transurethral resection of the bladder and at radical cystectomy. Tumour-infiltrating CD4^+^ lymphocytes were significantly hypomethylated in all four investigated lineage loci compared to CD4^+^ lymphocytes in lymph nodes and blood (lymph nodes vs tumour-infiltrating lymphocytes: *IFNG* -4229 bp *p* < 0.0001, *IL13 -*11 bp *p* < 0.05, *IL17A* -122 bp *p* < 0.01 and *FOXP3* -77 bp *p* > 0.05). Examination of individual lymph nodes displayed different methylation signatures, suggesting possible correlation with future survival. More advanced post-cystectomy tumour stages correlated significantly with increased methylation at the *IFNG* -4229 bp locus. Patients with complete response to neoadjuvant chemotherapy displayed significant hypomethylation in CD4^+^ T cells for all four investigated loci, most prominently in *IFNG p* < 0.0001. Neoadjuvant chemotherapy seemed to result in a relocation of Th1-committed CD4^+^ T cells from blood, presumably to the tumour, indicated by shifts in the methylation patterns, whereas no such shifts were seen for lineages corresponding to *IL13*, *IL17A* and *FOXP3*.

**Conclusion:**

Increased lineage commitment in CD4^+^ T cells, as determined by demethylation in predictive CpG sites, is associated with lower post-cystectomy tumour stage, complete response to neoadjuvant chemotherapy and overall better outcome, suggesting epigenetic profiling of CD4^+^ T cell lineages as a useful readout for clinical staging.

**Electronic supplementary material:**

The online version of this article (10.1186/s13148-018-0536-6) contains supplementary material, which is available to authorized users.

## Background

Urinary bladder cancer (UBC) is the ninth most frequent cancer disease with 380,000 new cases diagnosed worldwide and about 150,000 deaths yearly [1, 2]. Environmental factors and life style seem to play an important role for tumour development. Chronic exposure to carcinogenic substances in the urine such as smoking-derived carcinogens, rubber and certain dyes may lead to cancer development [3]. In addition, infection with the trematode *Schistosoma haematobium* leads to chronic inflammation in the urinary bladder and development of squamous cell carcinoma [4]. Thus, chronic exposure to irritating substances, i.e. chemicals or pathogens, may lead to malignant transformation of cells and finally cancer development. Urothelial muscle invasive bladder cancer is diagnosed (defined as tumour stages T2-T4aN0M0), based on the pathologist’s assessment of tumour obtained at transurethral resection of the bladder (TUR-B). Patients judged to be fit according to the Swedish national guidelines are treated with cisplatin-based neoadjuvant combination chemotherapy (NAC), prior to radical cystectomy (RC) typically MVAC (methotrexate, vinblastine, doxorubicin and cisplatin).

UBC development is highly associated with inflammation and immune cell infiltration, an association that provides a basis for immunotherapeutic strategies, such as intravesically administered BCG (Bacillus Calmette-Guerin vaccine) in treatment of high-risk non-muscle invasive bladder cancer (HR-NMIBC) [5]. We previously demonstrated that the presence of CD3^+^ tumour-infiltrating T lymphocytes (TIL) is a positive prognostic factor for survival [6], supporting the importance of an anti-tumour T cell response. We have also demonstrated that the regional lymph nodes (LNs) contain lymphocytes that are reactive towards the tumour [7, 8], but that the inter-patient variation of responsiveness to autologous tumour antigen stimulus is highly variable.

The maturation process of T lymphocytes is localized in the thymus through a process of positive and negative selection resulting in CD4^+^ MHC class II-restricted T cells and CD8^+^ MHC class I-restricted T cells [9]. Upon encounter of intermediate affinity/concentration of self-peptides in the thymic medulla, naïve CD4^+^ T cells are converted to Foxp3 stably expressing regulatory T cells (Treg). CD4^+^ T cells emerging from the thymus pass into the periphery and circulate various tissues. Upon encountering their cognate antigen in a tumour setting, the pattern of their maturation and differentiation into separate CD4^+^ T cell lineages will be decided by the combined signals from the antigen-presenting cells, tumour cells and stroma cells present in this distinct environment. The main CD4^+^ T cell effector lineages are Th1, Th2 and Th17, as recognized by their production of effector cytokines IFN-γ, IL-13 and IL-17A, respectively [10]. Upon activation and proliferation, naïve T cells transform to differentiated effector cells with a stable phenotype that is difficult to reverse after five cell divisions [11, 12]. However, plasticity among committed T cell subpopulations have started to be explored [13–15]. Long-term epigenetic stability of a T cell phenotype can be evaluated using methylation markers at predictive CpG sites [16–19].

We and other groups have previously investigated the methylation status of the *IFNG* locus in CD4^+^ T cells from LNs and tumours. We have also developed methods for investigating the *IL13*, *FOXP3* and *IL17A* loci to make a global CD4^+^ T cell assessment regarding epigenetic commitment [17, 19–21].

Based on previous experiences and results, we performed a snapshot analysis of the in vivo epigenetic commitment of CD4^+^ T cell populations in samples from patients with UBC, using DNA methylation pattern of epigenetic lineage markers for Th1, Th2, Th17 and Tregs predictive for assessing CD4^+^ T cell subpopulation stability [19]. Further, we correlate our findings with clinical response to neoadjuvant chemotherapy and pathological tumour stage post-cystectomy.

## Methods

### Patient inclusion and clinical procedure

All patients were included in this study after giving their written and oral consent to participate, in accordance with the declaration of Helsinki. The study was approved by the local ethical committee (dnr: 2007/71-31, amendment 2017/190-32). Recruitment was performed between 2014 and 2017 from nine Swedish hospitals (Umeå University Hospital, Sundsvall Hospital, Västerås Central Hospital, Linköping University Hospital, Norrköping Hospital, Skellefteå Hospital and Gävle Hospital, Uppsala Akademiska University Hospital and Östersund County Hospital). The patients in the study were included either before TUR-B with suspected urinary bladder cancer, or before RC, after an established muscle invasive bladder cancer (MIBC) (tumour stages cT2-4aN0M0), as determined by pathologist’s assessment of previously performed TUR-B. Patient characteristics are presented in Table 1. Tumour and blood samples were collected at TUR-B from 23 patients. Samples were also collected from 21 patients at RC, including blood, tumour draining sentinel lymph nodes (SN), non-draining lymph nodes (nSN) and, in cases with remaining tumour at this stage, tumour tissue. The method for sentinel node detection has previously been described [22]. From six patients, additional blood samples were obtained during NAC treatment (in-between NAC cycles). The total number of specimens was as follows: blood, *n* = 48; regional LN (both SN and nSN), *n* = 76; and tumour, *n* = 22. Not all specimens were analysed for every parameter, due to sample limitations.

**Table 1:** Patients included in this study

No.	Age	Gender	Sampling	Clinical T-staging	pT stage	NAC	NAC response	LN	Tumor	Year
1	73	M	T	cTaG2	–	–	–	–	Tumour	2017
2	81	F	T, C	cT3	pT3	–	–	4	Tumour	2017
3	60	F	C	cT2	pTa-pTis	NAC	PR	2	–	2017
4	70	M	T	cTaG3	–	–	–	–	Tumour	2017
5	82	M	T	cT3	–	–	–	–	Tumour	2017
6	73	M	C	cT2	pTa-pTis	–	–	3	Tumour	2017
7	74	M	C	cT2	pT3	NAC	prog.	3	–	2017
8	62	M	C	cT2	pT0	NAC	CR	5	–	2017
9	73	M	C	cT2	pT3	NAC	prog.	4	–	2017
10	57	F	C	cT2	pT0	NAC	CR	9	Tumour	2017
11	70	M	C	cT2	pT1	NAC	PR	2	–	2017
12	68	M	T	cT1	–	–	–	–	Tumour	2017
13	68	M	C	cT2	pT0	NAC	CR	6	–	2017
14	73	M	T	cT2	–	–	–	–	Tumour	2017
15	61	F	T	cTaG2	–	–	–	–	Tumour	2017
16	75	M	C	cT2	pT0	NAC	CR	1	–	2017
17	70	F	T	cTaG2	–	–	–	–	Tumour	2017
18	73	F	T, post Ch	cT3	–	NAC	–	–	Tumour	2017
19	64	F	T, C	cT2	pT2	NAC	NR	4	Tumour	2017
20	71	M	C	cT2	pT2	NAC	NR	7	–	2017
21	77	M	T	cT1	–	–	–	–	Tumour	2017
22	72	M	T, post Ch	cT2	–	NAC	–	–	Tumour	2017
23	61	M	T	cT1 + CIS	–	–	–	–	Tumour	2017
24	68	M	C	cT2	pT0	NAC	CR	2	–	2017
25	79	F	T	cT2	–	–	–	–	Tumour	2017
26	74	M	T	cTaG2	–	–	–	–	Tumour	2017
27	67	M	C	cT2	pTa-pTis	NAC	PR	4	–	2017
28	67	F	T	cT2	–	NAC	–	–	Tumour	2017
29	79	M	T	cT1	–	–	–	–	Tumour	2017
30	84	F	T	Benign	–	–	–		Tumour	2017
31	71	M	C	cT2	pT0	NAC	CR	5	–	2017
32	75	M	T	cTaG2	–	–	–	–	Tumour	2017
33	69	F	T, post Ch	cT4a	–	–	–	–	–	2017
34	50	M	T, C post Ch	cT2	pT0	NAC	CR	4	–	2015
35	56	M	C	cT2	pT0	NAC	CR	3	–	2015
36	79	M	C	cT3	pT3	NAC	NR	4	–	2015
37	59	F	T	cT3	–	–	–	–	Tumour	2017
38	80	M	C	cT2	pT0	–	–	4	–	2017
39	60	M	T, C post Ch	cT3	–	NAC	prog.	–	–	2014
40	66	M	T, C post Ch	cT3	–	NAC	CR	–	–	2017

NAC was administered to indicated patients

*Age*, in years at time of inclusion; gender, *M* male, *F* female; sampling, *T* TUR-B, *C* radical cystectomy, *Ch* during chemo blood (post-chemo); NAC responder, *CR* complete responder, *NR* non-responder, *PR* partial responder, *prog*. progression; *LN*, number of lymph nodes obtained indicated, *Tumour*, specimens acquired from indicated patients. *Year*, years of intervention. –, no data/sample available

### Lymphocyte extraction

Peripheral blood mononuclear cells (PBMCs) from blood were extracted using Ficoll paque PLUS (GE Healthcare).

Tumour-infiltrating lymphocytes were extracted by cutting tumour tissue into small pieces and disassociating the dissected tumour pieces into single cells using a gentleMACS dissociator (Miltenyi Biotec). Samples were processed in GIBCO Aim V™ (Invitrogen) supplemented with collagenase/hyaluronidase (STEMCELL Technologies). Subsequently, the single cell suspension was strained through a 40-μm strainer to exclude remaining tumour cell aggregates and tissue debris.

Lymph nodes were gently homogenized by straining through a 40-μm strainer.

### CD4^+^ T lymphocyte purification

**Fig. 1:**
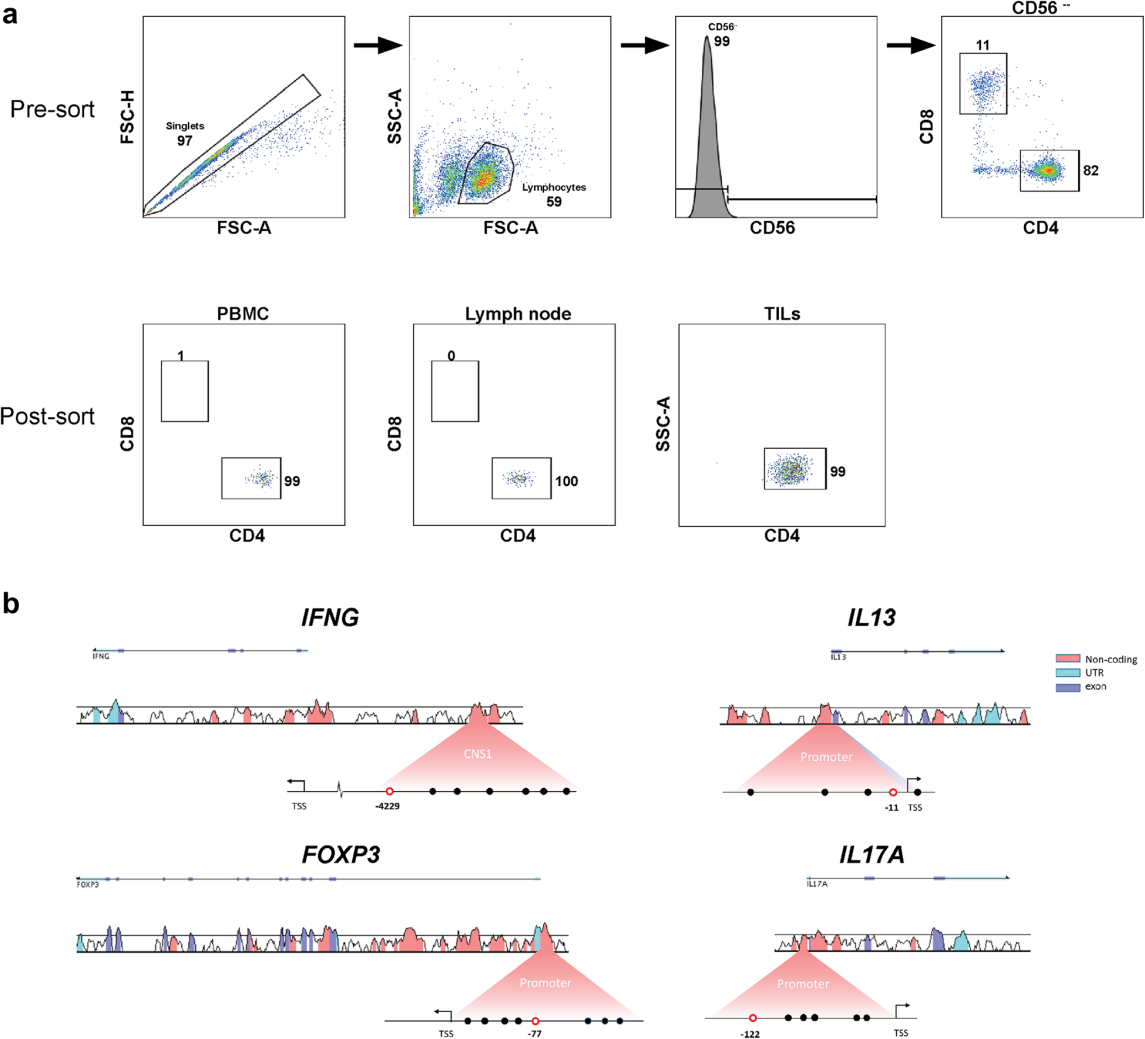
Sorting strategy and loci visualization. **a** CD3^+^ cells derived from PBMC, lymph nodes or tumour tissue were sorted by flow cytometry using a FACSARIA. Gating strategy was done as follows: singlets > lymphocytes > CD56^−^ > CD4^+^. CD4^+^CD56^−^. Purity analysis is presented in percentage of parent. Plots show representative best. **b** Signature loci visualized by VISTA-plots. The conservation of the assessed gene loci between human and mouse is depicted relative to the genes. The conservation is defined as stretches of nucleotides more than 100 bp with over 70% conserved area. The magnified area schematically describes the investigated regions. Analysed CpG is marked as red circle, describing its position in relation to transcription start site (TSS). *CNS1* conserved non-coding sequence 1, *UTR* untranslated region

CD4^+^ T lymphocytes from PBMC, lymph nodes and tumours were sorted in two steps: (1) pre-sorting of total CD3^+^ cells was performed using EasySep Human CD3-positive selection kit II (STEMCELL Technologies). CD3^+^ pre-sorted cells were then stained with anti-human CD4 (PerCp Cy5.5, BioLegend), anti-human CD8 (APC, BD Biosciences), anti-human CD56 (PE, BD Biosciences), anti-human CD45RA (V500, BD Biosciences) and anti-human C45RO (APC CY7 BioLegend). (2) CD4^+^ cells were FACS-purified according to gating strategy presented in Fig. 1a using a BD FACSARIA I instrument (BD Biosciences). The purity of CD4^+^ T lymphocytes was confirmed post-sorting and was consistently ≥ 95%. Analysis of flow cytometry data was performed using the FACS Diva software (BD Biosciences) and FlowJo (version 10, FlowJo LLC). Sorted cells were pelleted and stored in − 20 °C until further analysis.

### Pyrosequencing of CD4^+^ lymphocytes

DNA from CD4^+^ cell pellets were extracted and bisulfite converted using EZ DNA methylation Direct kit (Zymo Research). The locus-specific pyrosequencing PCRs for *FOXP3*, *IFNG IL13* and *IL17A* were conducted using primers where one primer for each PCR reaction was biotinylated (Thermo Scientific and Biomers.net) (Additional file 1: Table S1). The PCRs were performed using a Thermal cycler (Bio-Rad). The PCR product was immobilized using the PyroMark Q96 vacuum workstation (Qiagen). The sequencing reaction was performed on a Pyro Q96, using Pyromark Gold 96Q reagents (Qiagen) and PCR assay-specific sequencing primers (Additional file 1: Table S1). Analysis of the sequence data were performed by the Pyromark Q96 ID software (Qiagen) giving individual percentage for assayed CpGs. Graphic visualization of the four loci (Fig. 1b) was made using VISTA-point [23]. Histograms demonstrate species conservation between human and mouse, and circles below schematically demonstrate CpG sites in the specific region. The analysed signature CpG site is marked in red with their location, in base pairs, from transcription start site (TSS) indicated.

### Cisplatin cultures

CD4^+^ T lymphocytes were extracted from peripheral blood of healthy donors (*n* = 4) as described above. Cells were put in cultures at a concentration of 1 × 10^6^ cells/ml in GIBCO Aim V™ medium, research grade (Invitrogen) supplemented with l-glutamine (Sigma-Aldrich). Cells were stimulated at day 0 or day 6, with 5 μg/ml plate bound αCD3 (BioLegend) and 2 μg/ml soluble αCD28 (BioLegend). Cisplatin (Hospira, Pfizer) was added on day 0 or day 6 of culture at a concentration of 25 μM (LD_50_ [24], for subsequent ELISA) or 50 μM (for subsequent pyrosequencing). All cultures were incubated at 37 °C in 5% CO_2_. Cells were harvested at day 12.

### 5-Methylcytosine ELISA

DNA from cultures exposed to 25 μM cisplatin was extracted using DNeasy blood and tissue kit (Qiagen). DNA quantity and quality were measured on a Nanodrop instrument (ThermoFisher Scientific), and 100 ng DNA/well was utilized to perform ELISA. 5-Methylcytosine (5mC) ELISA was conducted, and each sample was run in duplicate, using 5-mC DNA ELISA kit (ZYMO research) according to the manufacturer’s instructions. Sample values were normalized against either day 0 untreated samples or day 12 samples without cisplatin treatment.

### Statistical analysis

All statistical analysis was performed using GraphPad PRISM version 7.04. Non-parametric Mann-Whitney test, Kruskal-Wallis test and Friedmans test was used where applicable. Dunn’s multiple comparisons test was employed when suitable. Statistical significance in graphs are shown as * if *p* < 0.05, ** if *p* < 0.01, *** if *p* < 0.001 and **** if *p* < 0.0001. Plots show mean with standard error of mean (SEM). No statistical calculations were made on data in Fig. 8a, c, d due to low sample number. Groups of data were consistently excluded from statistical analysis if *n* = < 4.

## Results

Samples from patients with urinary bladder cancer, presented in Table 1, were analysed. Single cell suspensions from PBMC, tumour and lymph nodes were stained and sorted by flow cytometry (Fig. 1a). FACS analysis post-sorting demonstrated ≥ 95% purity (Fig. 1a). DNA was extracted and bisulfite converted for pyrosequencing and assessment of signature CpG sites for analysis of lineage commitment to the Th1, Th2, Treg and Th17 lineages, using previously identified predictive sites in the corresponding genes *IFNG*, *IL13*, *FOXP3* and *IL17A* (Fig. 1b) [17, 19–21].

### Evaluation of CD4^+^ T cell lineage commitment

**Fig. 2:**
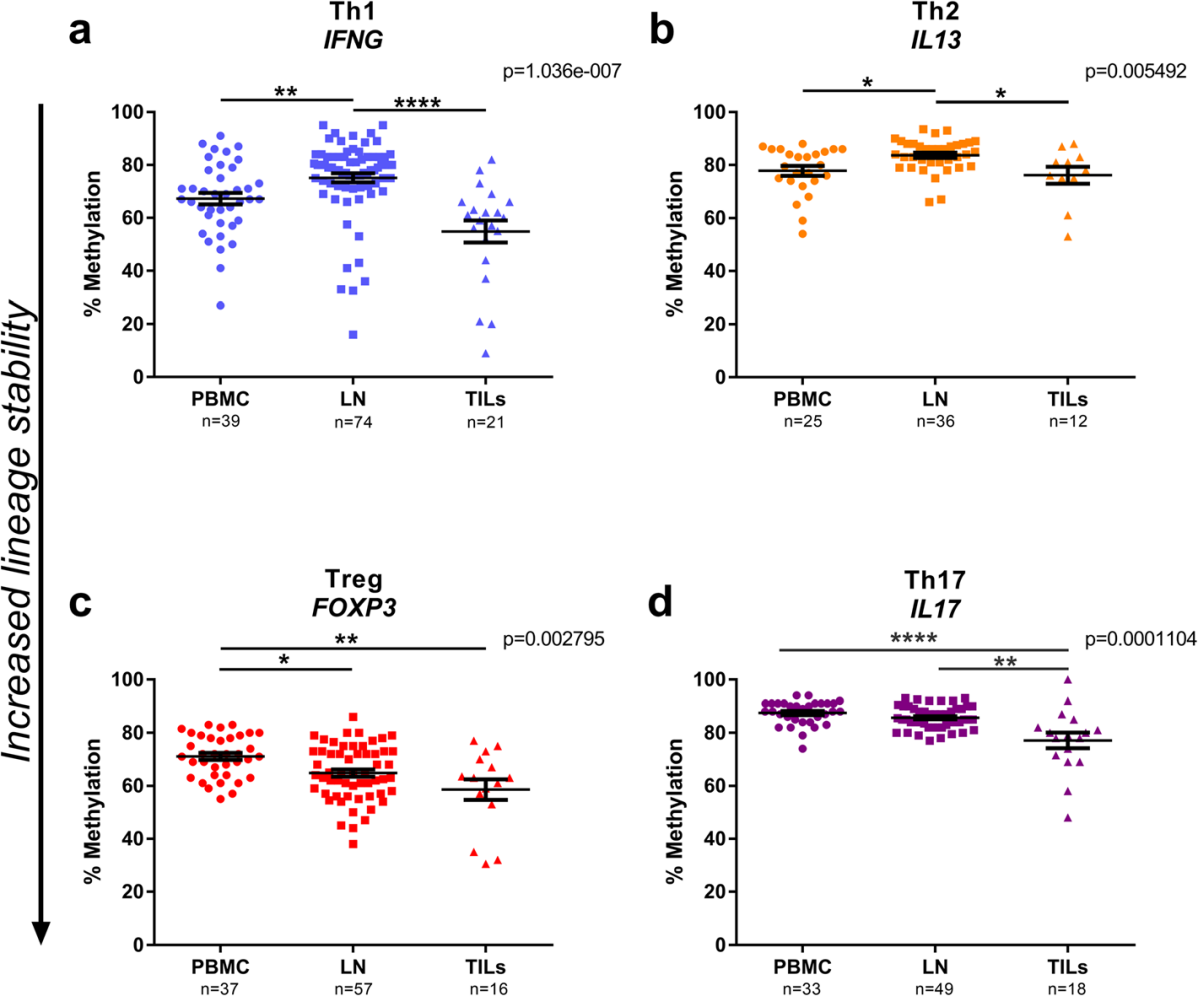
CD4^+^ T cell lineage commitment in urinary bladder cancer. CD4^+^ T cells sorted from PBMC, LN and TILs were analysed by pyrosequencing at CD4^+^ T cell signature loci: **a** *IFNG* for Th1, **b** *IL13* for Th2, **c** *FOXP3* for Treg and **d** *IL17A* for Th17. *p* values stated in graphs are generated from Kruskal-Wallis test. *p* values from Dunn’s multiple comparisons test are indicated as *p < 0.05, **p < 0.01, ***p < 0.001 and ****p < 0.0001. Plots show percentage of methylation for every specimen analysed (*n* stated on *x*-axis beneath sample type). Bars indicate mean with error bars displaying SEM. Downward arrow along *y*-axis illustrate increased lineage commitment, as a result of decreased methylation

The four selected loci were investigated in CD4^+^ T lymphocytes sorted from PBMCs, LNs and tumours obtained at TUR-B and RC. Comparisons of sentinel and non-sentinel lymph node data demonstrated no significant differences (data not shown), and thus, these specimens were analysed together as a group referred to as “lymph nodes” (LN) throughout the analysis. The *IFNG* locus methylation in the CpG position -4229 bp from transcription start site (TSS) (located in the conserved non-coding sequence 1 (CNS1)) (Fig. 1b) was used to assess the Th1-committed CD4^+^ T cells [20]. CD4^+^ T cells from TILs were significantly more demethylated in the *IFNG* locus compared to LN (*p* < 0.0001), whereas the methylation in LNs were higher compared to PBMC, suggesting an increased infiltration of Th1 IFN-γ producing CD4^+^ T cells into the tumour (Fig. 2a). With regard to Th2-committed CD4^+^ T cells, the *IL13* locus was evaluated at the signature CpG position -11 bp from TSS as previously demonstrated [21] (Fig. 1b). Again, we found a significantly decreased level of methylation in the *IL13* locus in TILs compared to lymph nodes (*p* < 0.05) (Fig. 2b). However, there were no significant changes when comparing CD4^+^ T cells derived from TILs with those from PBMCs. Interestingly, CD4^+^ T cells from LN demonstrated significantly increased methylation in the *IL13* locus compared with both PBMC (*p* < 0.05) and TILs (*p* < 0.05). CD4^+^ Tregs were assessed at the CpG -77 bp from TSS (Fig. 1b) in the *FOXP3* locus as previously described [17]. This signature CpG was significantly hypomethylated in CD4^+^ T cells from lymph nodes (*p* < 0.05) and tumours (*p* < 0.01) compared to PBMC (Fig. 2c). Finally, Th17 cells were investigated at the *IL17A* signature locus at the CpG -122 bp from TSS (Fig. 1b) [19]. CD4^+^ T cells from tumours demonstrated significantly decreased methylation compared to LN (*p* < 0.05) and PBMCs (*p* < 0.0001) (Fig. 2d). No significant difference was seen when comparing CD4^+^ T cells from PBMC derived from either TUR-B or RC, rationalizing the equivalence between the samples obtained at the two time points (Additional file 2: Figure S2).

### Analysis of lineage commitment at the time of TUR-B

**Fig. 3:**
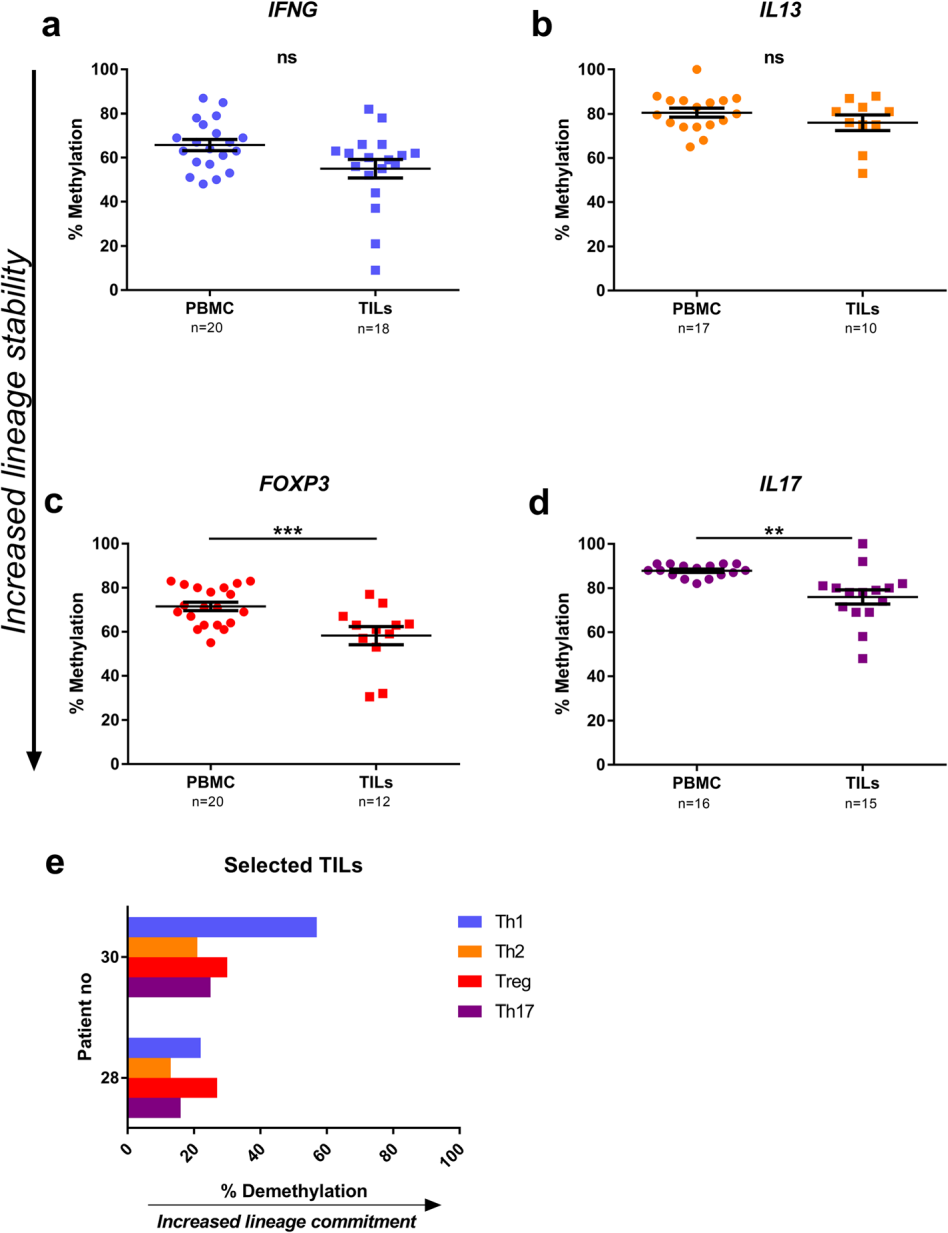
DNA methylation analysis of CD4^+^ T cells from PBMC and TILs at time of TUR-B. Methylation levels of **a** *IFNG*, **b** *IL13*, **c** *FOXP3* and **d** *IL17A* signature loci in CD4^+^ T cells from PBMC and TILs were analysed by pyrosequencing at time of TUR-B. Plots show percentage of methylation for every specimen analysed (*n* stated on *x*-axis beneath sample type). *p* values are indicated as *p < 0.05, **p < 0.01, ***p < 0.001 and ****p < 0.0001, using Kruskal-Wallis test. Bars indicate mean with error bars displaying SEM. Downward arrow along *y*-axis illustrate increased lineage commitment, as a result of decreased methylation. **e** Bar graphs depicting demethylation levels at the four signature sites (Th1 = *IFNG*, Th2 = *IL13*, Treg = *FOXP3*, Th17 = *IL17A*) from two patients, selected for their low vs high methylation pattern in *IFNG*. TILs from patient no. 30 with low methylation levels in *IFNG* (top bars) and patient no. 28 with high methylation in *IFNG*

Patient materials obtained at the time of TUR-B were analysed separately. CD4^+^ T cells purified from fresh TUR-B tumour resections did not demonstrate any difference in methylation profile in the *IFNG* locus compared to PBMCs (*p* > 0.05) (Fig. 3a). Neither was there any difference in the degree of methylation in the *IL13* locus (Fig. 3b). With regard to Tregs, the *FOXP3* signature locus was significantly demethylated in CD4^+^ T cells from the tumour compared to PBMCs (*p* < 0.001) (Fig. 3c). Finally, we found a decreased methylation of the *IL17A* locus in CD4^+^ T cells in the tumour compared to PBMCs (*p* < 0.01) (Fig. 3d). One patient (no. 30), included at TUR-B had benign disease and was hence not included in the general data analysis. We examined the TILs from this patient and found the *IFNG* locus to be highly demethylated, whereas the three other loci were hypermethylated, suggesting a Th1 lineage commitment (Fig. 3e, top bars). For comparison, we examined TILs from a patient (no. 28) with hypermethylation at the *IFNG* locus (Fig. 3e bottom bars). This patient (cT stage cT2a) displayed hypermethylation in all four loci, corresponding to an overall low lineage differentiation.

### Analysis of lineage commitment at the time of cystectomy

**Fig. 4:**
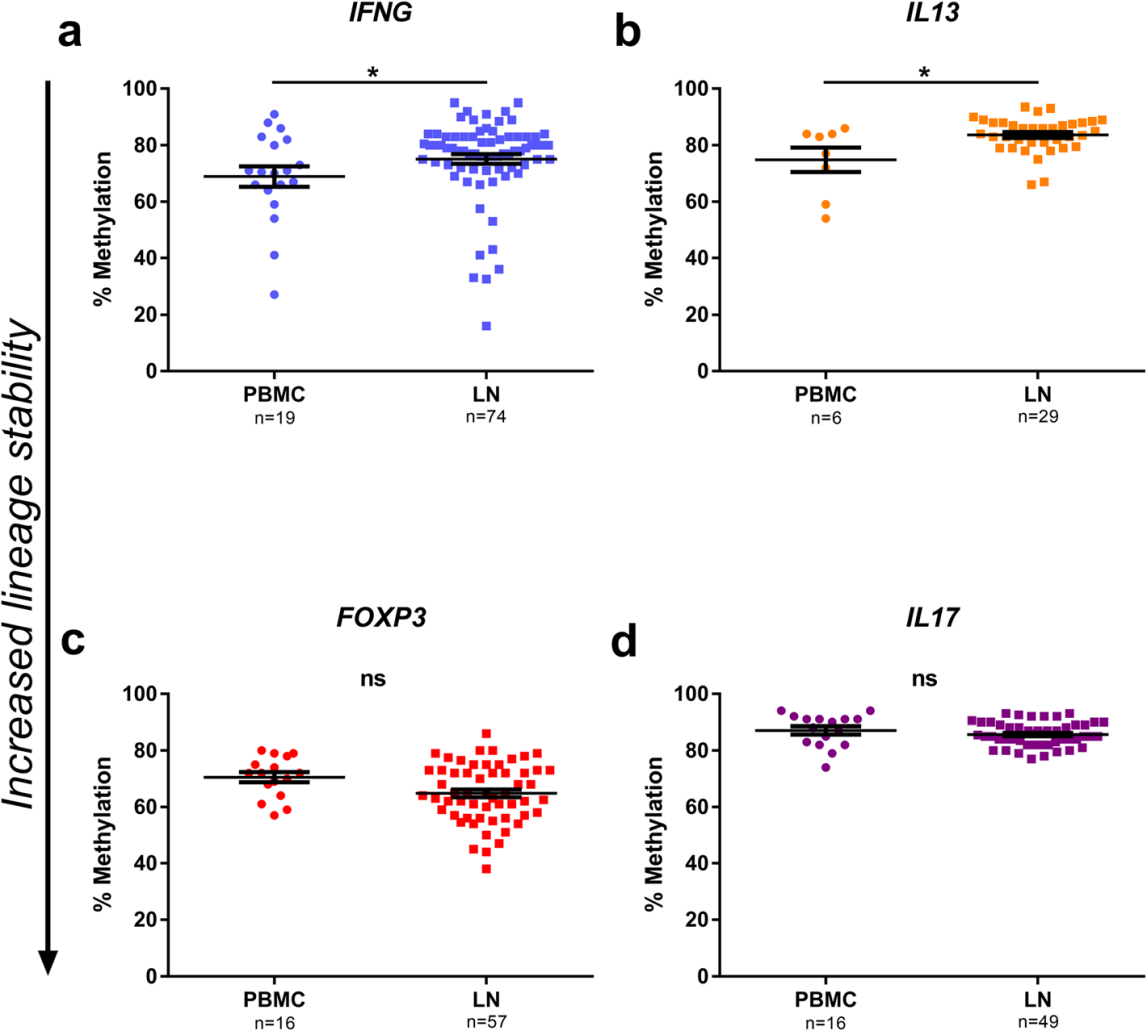
Methylation analysis in specimens from radical cystectomy (RC). Methylation percentage in CD4^+^ T cells from PBMC and lymph nodes retrieved at time of RC. *IFNG* (**a**), *IL13* (**b**), *FOXP3* (**c**) and *IL17A* (**d**) were analysed. Plots show percentage of methylation for every specimen analysed (*n* stated on *x*-axis beneath sample type). *p* values are indicated as *p < 0.05, **p < 0.01, ***p < 0.001 and ****p < 0.0001, using Mann-Whitney test. Bars indicate mean with error bars displaying SEM. Downward arrow along *y*-axis illustrate increased lineage commitment, as a result of decreased methylation

CD4^+^ T cells derived from samples obtained at the time of RC were analysed at the four signature loci. In LN, the methylation at the *IFNG* locus was significantly increased, compared to the corresponding cells from PBMC (*p* < 0.05) (Fig. 4a). Similarly, the level of methylation was increased at the *IL13* locus in lymph nodes compared to blood (*p* < 0.05) (Fig. 4b). No differences were seen in the methylation levels of *FOXP3* or *IL17A* loci between CD4^+^ T cells derived from PBMC and LN (Fig. 4c, d).

**Fig. 5:**
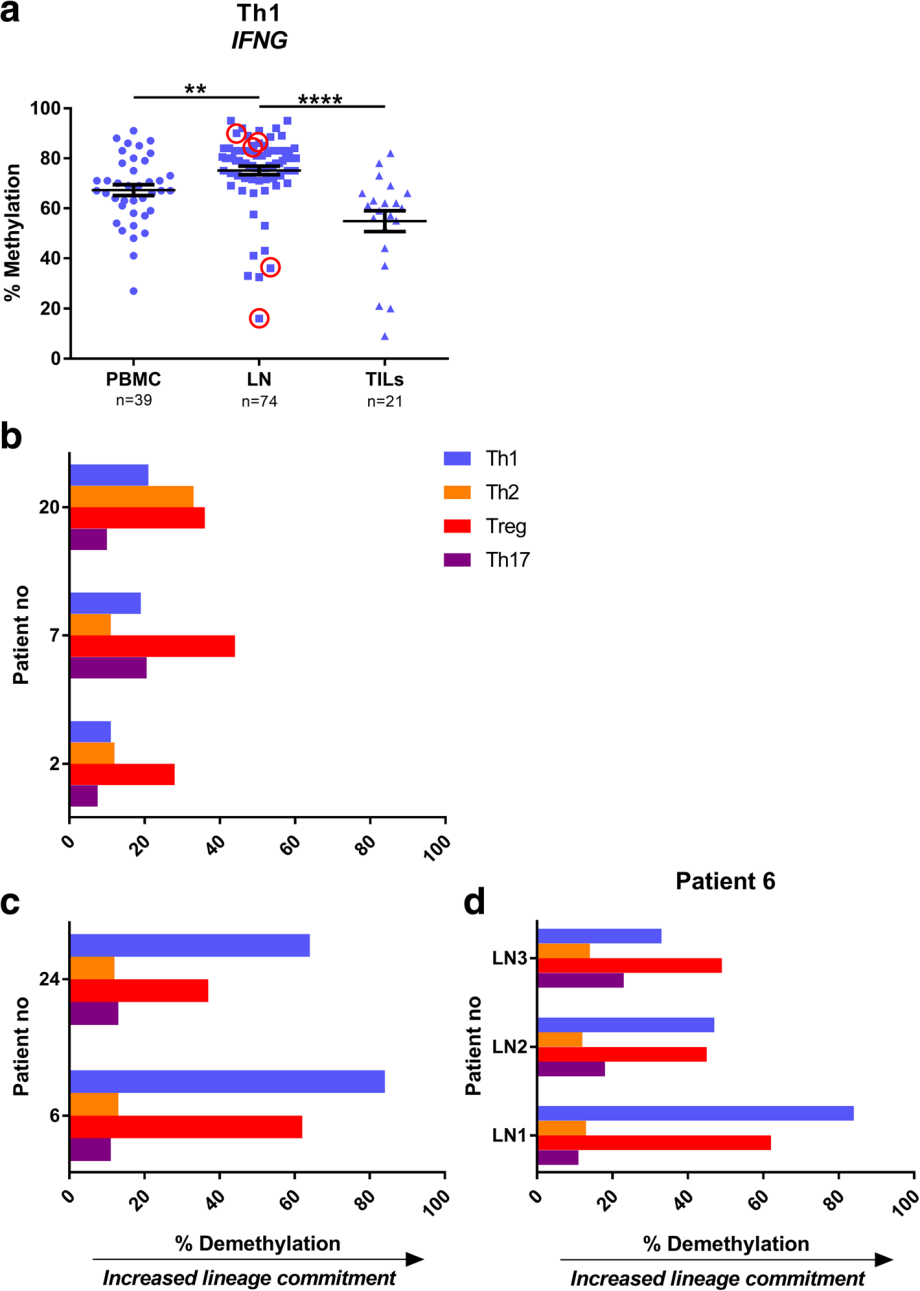
Case studies of lymph nodes with low or high methylation in *IFNG* locus. **a** Same as Fig. 2a, with evaluated lymph nodes circled in red. **b**–**d** Bar graphs depicts percentage of demethylation levels at the four signature sites, representing increase in lineage commitment. Th1 = *IFNG*, Th2 = *IL13*, Treg = *FOXP3*, Th17 = *IL17A*. **b** LN from three patients with high *IFNG* methylation. **c** LNs from two patients with low *IFNG* methylation patterns. **d** Case study of three LNs from patient no. 6, chosen for a distinct *IFNG* demethylation in one node (from Fig. 5c, lower bars)

CD4^+^ T cells from five lymph nodes (indicated by red circles in Fig. 5a) were selected for their high (Fig. 5b) or low (Fig. 5c) methylation profiles at the *IFNG* locus and were individually investigated for all four signature loci. The two specimens with low methylation at the *IFNG* locus also demonstrated a demethylated pattern in the Treg locus, while no signs of Th2 and Th17 skewing were found: i.e. *IL13* and *IL17A* signature loci were almost completely methylated (Fig. 5c, patient no. 6 and 24). On the contrary, the samples demonstrating a high methylation pattern in the *IFNG* locus displayed more of a Treg/Th2 or Treg/Th17 commitment judged by the methylation profiles in signature loci (Fig. 5b, patient no. 7 and 2). One LN revealed low commitment for all four loci compared to the other four LNs investigated (Fig. 5b, patient no. 20). The patients with LNs displaying low *IFNG* methylation, and therefore a Th1 signature, had a lower pathological tumour staging (pT stage), pTa-TisN1 (patient 6) and pT0 (patient 24) (Fig. 5c), although the former had a node metastasis (not included in specimens). The patients with LNs highly methylated at the *IFNG* locus had a more advanced disease stage, pT3aN2, pT3a and pT2a, respectively (patient, 7, 2 and 20) (Fig. 5b).

In order to epigenetically stage the collective immune response in a single patient (no. 6), the CD4^+^ T cell compartment in three individual LNs were analysed (Fig. 5d). LN1 demonstrated a Th1/Treg pattern, whereas the other two LNs (2 and 3) displayed different degrees of Treg/Th17 skewing, although still with a fraction of Th1commitment (Fig. 5d).

### CD4^+^ T cells from LN are differentially committed when stratified over pT stage

**Fig. 6:**
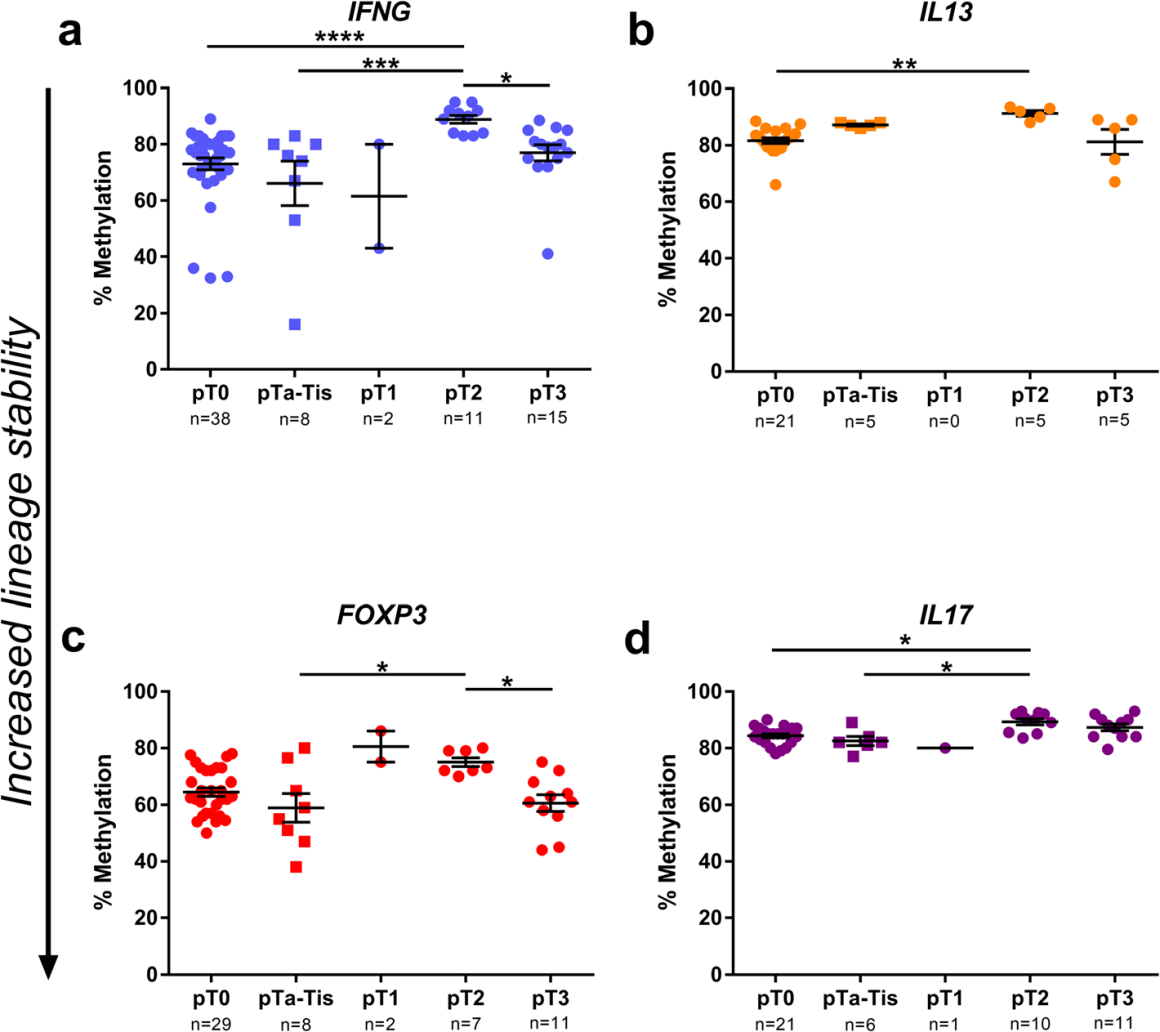
LN-derived CD4^+^ T cells stratified for pT stage. CD4^+^ T cells from lymph nodes stratified according to the patients’ pT stage. Plots show percentage of methylation at signature CpG, evaluated by pyrosequencing, for every lymph node analysed. Lymph node number (*n*) is stated on the *x*-axis beneath sample type. Group pT1 was excluded throughout all statistical analysis due to low sample number. **a** *IFNG* locus (Kruskal-Wallis test *p* < 0.0001), **b** *IL13* locus (Kruskal-Wallis test *p* < 0.001), **c** *FOXP3* locus (Kruskal-Wallis test *p* < 0.05) and **d** *IL17A* locus (Kruskal-Wallis test *p* < 0.01). *p* values from Dunn’s multiple comparisons test are indicated as *p < 0.05, **p < 0.01, ***p < 0.001, ****p < 0.0001. Bars indicate mean with error bars displaying SEM. Downward arrow along *y*-axis illustrates increased lineage commitment, as a result of decreased methylation

The pT stage determined by histopathology following cystectomy is known to predict prognosis and to function as a surrogate marker for overall survival in MIBC patients undergoing NAC [25]. Complete response (CR), i.e. pT0N0M0 at RC, corresponds to an excellent long-term survival. We evaluated the lineage commitment in the four loci according to pT staging. The *IFNG* locus demonstrated a demethylated pattern in the primary tumours (at TUR-B) of pT0 patients (complete NAC-responders) and in non-invasive tumours (pTa-Tis) compared to in primary tumours with muscle invasive tumour outcomes post-RC (pT2) (*p* < 0.0001 resp. *p* < 0.001) (Fig. 6a). The methylation was decreased in the perivesical infiltrating tumours (pT3) compared to the muscle invasive pT2 tumours (*p* < 0.05). In the *IL13* locus, the CD4^+^ T cells demonstrated an increased methylation in muscle invasive pT2 tumours compared to the cells from patients with pT0 stage (Fig. 6b). Methylation levels at the *FOXP3* locus was increased in LN CD4^+^ T cells from patient with muscle invasive pT2 compared to both non-muscle invasive pTa-Tis staged patients and to the cells from patients with perivesical infiltrating tumours (pT3) (*p* < 0.05 for both) (Fig. 6c). In *IL17A*, the methylation was increased in LN-derived CD4^+^ T cells from patients staged with pT2 compared to the pT0 as well as non-muscle invasive pTa-Tis staged patients (*p* < 0.05 for both) (Fig. 6d).

### Methylation patterns in lymph nodes correspond with response to neoadjuvant chemotherapy

**Fig. 7:**
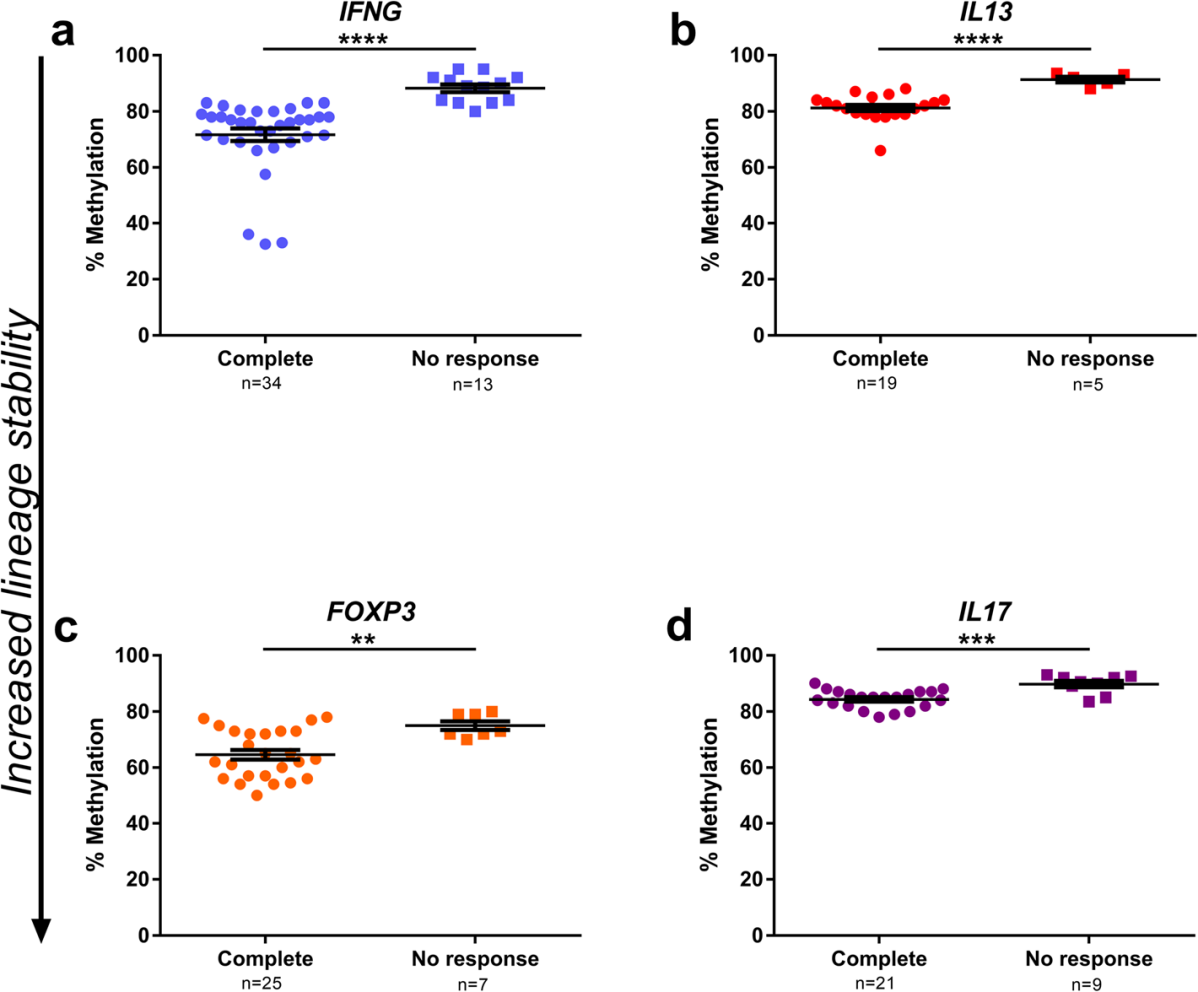
Lymph nodes grouped according to NAC response. CD4^+^ T cells from lymph nodes sorted according to the patients’ responses to neoadjuvant chemotherapy (NAC); complete response (CR; pT0N0M0) or no response (NR; pT2 or higher N0M0) Plots show percentage of methylation at signature CpG, evaluated by pyrosequencing, for every lymph node analysed. Lymph node number (*n*) stated on *x*-axis beneath sample type. **a** *IFNG* (number of patients: CR *n* = 8, NR *n* = 3). **b** *IL13* (number of patients: CR *n* = 4, NR *n* = 2). **c** *FOXP3* (number of patients: CR *n* = 6, NR *n* = 2). **d** *IL17A* (number of patients: *IL17A* CR *n* = 6 NR *n* = 2). Mann-Whitney test was used for the statistical analysis. *p* values are indicated as *p < 0.05, **p < 0.01, ***p < 0.001 and ****p < 0.0001. Bars indicate mean with error bars displaying SEM

Patients were stratified according to their clinical response to NAC, and LN-derived CD4^+^ T cells from patients with complete response (CR; pT0N0M0) and those with no response (NR; pT ≥ 2N0M0) were compared. Methylation was significantly lower in the group with CR compared to those with NR in all four loci, with the most pronounced difference in the *IFNG* locus (*IFNG p* < 0.0001, *IL13 p* < 0.0001, *FOXP3 p* < 0.01, *IL17A p* < 0.001) (Fig. 7a–d). When stratifying PBMC or LN samples according to their corresponding clinical T stage, it revealed no significant differences (data not shown).

### Th1 lineage affected during NAC treatment

**Fig. 8:**
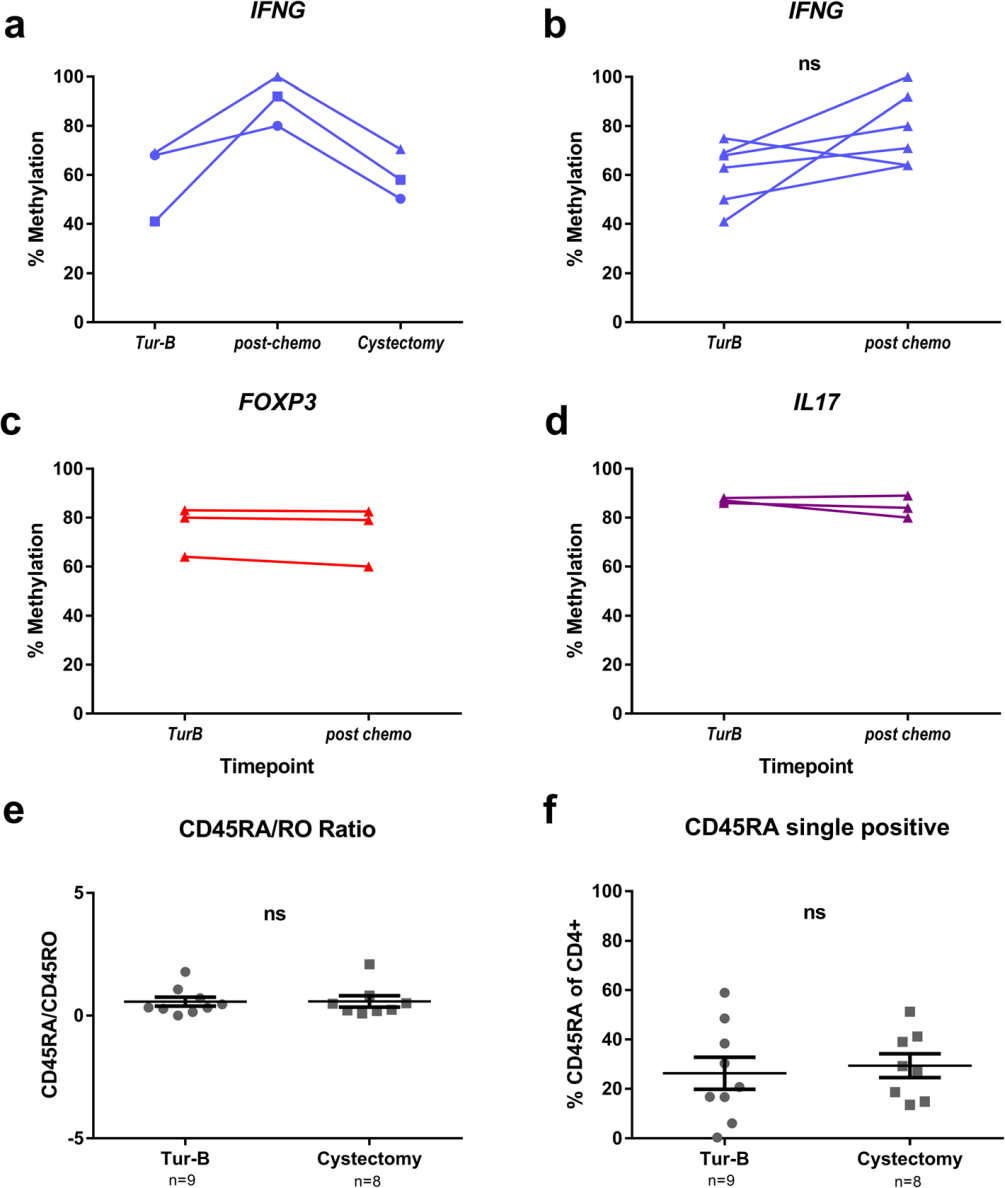
Investigation of sample retrieved during NAC treatment. **a** Examination of *IFNG* locus in CD4^+^ T cells from blood of three patients, taken at three time points: before (TUR-B), during (post-chemo) and after (cystectomy) neoadjuvant chemotherapy. **b** Methylation pattern in *IFNG* locus in paired samples from two time points, before (TUR-B) and during (post-chemo) NAC treatment (*n* = 6). Wilcoxon test was used for statistical analysis. **c** Methylation pattern in *FOXP3* locus in paired samples from two time points, before (TUR-B) and during (post-chemo) NAC treatment (*n* = 3). **d** Methylation pattern in *IL17A* locus in paired samples from two time points, before (TUR-B) and during (post-chemo) NAC treatment (*n* = 3). **e** CD45RA/RO protein expression ratio in PBMC derived CD4^+^ T cells from TUR-B or cystectomy, as evaluated by flow cytometry. **f** Percentage of CD45RA^+^ CD45RO^−^ (single positive) CD4^+^ T cells, determined by flow cytometry, at the two time points TUR-B and RC. **e**, **f** (*n* stated on *x*-axis beneath sample type) Bars show SEM

Patients with MIBC, and in WHO class 0–1 health condition, are according to the national Swedish guidelines recommended to receive 3–4 cycles of cisplatin-based NAC prior to RC. Blood samples were obtained after first or second cycle of NAC therapy and CD4^+^ T cells from PBMCs were extracted. Analysis of *IFNG* locus methylation at TUR-B, during NAC and at the time of RC demonstrated a temporary increase in the methylation status (*n* = 3) during NAC treatment (Fig. 8a). When comparing PBMC derived CD4^+^ T lymphocytes from TUR-B with corresponding cells obtained during chemotherapy for an additional three patients, we found a tendency to increase methylation in the *IFNG* locus, however not significant (Fig. 8b). Interestingly, when investigating the *FOXP3* and *IL17A* loci at the same time points, no change in methylation status was noted (Fig. 8c, d). To investigate if the change in the *IFNG* locus methylation signature was altered due to a recruitment of naïve T cells into the circulation, we calculated the ratio between naïve CD45RA^+^CD45RO^−^ and memory CD45RO^+^ T cells. We found no significant difference in the CD45RA/CD45RO ratio or in the fraction of CD45RA single positive cells between TUR-B and cystectomy (after NAC) samples (Fig. 8e, f), suggesting that the hypermethylation of the *IFNG* locus is not due to an increased inflow of naïve CD4^+^ T cells into the circulation (Fig. 8a).

### Cisplatin does not affect the DNA methylation

Cisplatin is the base of the neoadjuvant treatment, acting partly through cross binding of DNA (purine bases) [26, 27]. To investigate the possibility that cisplatin affects DNA methylation, in vitro cultures were established using CD4^+^ T lymphocytes from healthy donors (*n* = 4). 5mC ELISA showed no significant difference in the global methylation of cells treated with cisplatin (Additional file 3: Figure S1a). In addition, pyrosequencing of the *IFNG* locus revealed no significant difference in the site-specific methylation (Additional file 3: Figure S1b).

## Discussion

We here demonstrate that valuable information can be obtained by studying signature CpG methylation, indicating the degree of lineage commitment of CD4^+^ T cells in tissues from UBC patients. CD4^+^ T cell lineage commitment was found to be more pronounced in tumour-infiltrating lymphocytes (TILs) compared to PBMC and regional lymph nodes, indicating that differences in the tissue environments have a significant impact on CD4^+^ T cell destiny. In addition, we found correlations between increased lineage commitment of CD4^+^ T cells in LNs after neoadjuvant chemotherapy and an improved prognosis, indicating an important role for T cell immunity in the evolution of UBC towards more aggressive forms. To the best of our knowledge, this is the first time DNA methylation of CD4^+^ T cell lineage markers has been investigated in cells harvested from patients with UBC, and our data suggests that this type of investigation can contribute towards a deeper understanding of the role of the immune system in the UBC setting.

As a method, methylation analysis has advantages when compared to analysis of protein and mRNA expression. No stimulation of the cells is required in order to analyse the current methylation status; and thus, resting unmanipulated cells can be analysed for phenotype stability, with no risk of misinterpreting temporary, transient protein expression for stable effector lineages. This is a clear advantage when examining primary cells from clinical specimens since no tampering of the cells is needed. However, epigenetic status does not convey if the cells are active or resting, but rather indicate lineage commitment and effector capacity of the cells upon activation.

We demonstrate that TILs have a high degree of CD4^+^ T cells with lineage commitment (Fig. 2), proposing an active immune response towards the tumour. The *IFNG-*committed Th1 compartment is the most prominent of the four T cell lineages examined here, indicating that Th1 is the major lineage response towards the tumour, which is in agreement with the literature [28]. Furthermore, the methylation pattern in the *IFNG* locus has the greatest variability, compared to the other three loci, in all investigated tissues, which we interpret as both intra- and inter-patient variation (Fig. 2). The Treg compartment was the only lineage with a gradual decrease in methylation, with the highest methylation in blood, through lymph nodes to the lowest methylation in tumour. This could be interpreted as an effect of the immune-stimulatory environment, where high proliferation rate leads to an increase in the Treg population [6]. In our patient samples, we assumed that a prolonged immune response towards the tumour would be present, and this seemed to lead to a co-commitment of several stable lineages, mainly Th1 and Tregs (Figs. 3 and 5b, c). It becomes evident, when individually examining the four lineages in separate samples, that the specimens low in *IFNG* methylation had a clear Th1/Treg profile in both TILs and LN (Figs. 3e top bars and 5c), whereas the highly methylated specimens inclined more towards a Th2 or Th17 profile (Figs. 3e bottom bars and 5b), or an overall low lineage commitment pattern (Fig. 5b, patient 2). Noteworthy, the specimen with the most prominent demethylation in *IFNG* locus had a benign tumour, suggesting a protective IFN-γ response limiting the progress of the tumour (Fig. 3c top bars).

When separating the material according to the two time points of intervention (TUR-B and RC), the CD4^+^ T cells were found to display a higher degree of lineage commitment in TILs from the TUR-B (Fig. 3) compared to those from blood, while the LN CD4^+^ T cells at the time of cystectomy showed less lineage commitment or no difference compared to their blood counterparts (Fig. 4). We suggest that this is due to the selective migration of activated T cells away from the LN towards the tumour.

Stratifying CD4^+^ T cells from LNs according to the patients’ pT stage revealed differences in methylation state relating to local tumour responses to NAC, which is correlated with disease progression. The level of committed hypomethylated CD4^+^ T cells were increased in patients with low, non-muscle invasive pT stages as compared to those from patients with muscle invasive pT2 tumours (Fig. 6), which was most prominent in Th1 cells. These data suggest that an increase in committed Th1 effector cells in the tumour region is favourable for prognosis. Surprisingly, both Th1 and Treg commitment is increased in pT3, when the tumours have progressed to perivesical infiltration (a clear sign of a lack of response to NAC), perhaps suggesting that the balance between regulation and effector function has switched in the environment of more aggressive tumour cells.

The response to NAC in terms of histopathological tumour regression is a major positive prognostic factor for overall survival following radical cystectomy [25, 29]. Therefore, it was striking that the lineage commitment in LNs from patients with CR after NAC was significantly higher than in those not responding as favourably (Fig. 7). Our group has also previously demonstrated that cisplatin-based NAC induces immune-stimulatory effects [24, 30] and the present findings are in line with that context.

The lack of paired patient samples from TUR-B and RC prevented us from investigating the methylation pattern in pre-treatment TILs from the patients who received NAC and correlate to subsequent NAC response in corresponding LNs. Instead, we examined the methylation pattern in paired blood samples, obtained during the course of NAC treatment. During the NAC treatment (post-chemo), PBMC-derived CD4^+^ T cells demonstrated a tendency towards increased methylation in the *IFNG* locus (Fig. 8a, b) compared to paired blood samples acquired before NAC (at time of TUR-B) as well as after NAC (at time of RC). There was no indication of changes in *FOXP3*, *IL17A* or *IL13* methylation between these time points (Figs. 7c and 8d, data not shown). We further establish that the changes in DNA methylation are not a direct effect of cisplatin, by performing both a whole genome 5mC ELISA to ensure that this agent does not affect methylation on a global level, and an *IFNG* locus-specific analysis, for comparison with our data (Additional file 3: Figure S1). Since the CD45RA/CD45RO ratio between the two occasions of TUR-B and RC was not changed (Fig. 7e, f), we propose that the temporary increase of methylation in the *IFNG* locus is not due to the recruitment of naïve cells, but rather to the migration/relocation specifically of Th1 lineage-committed T cells towards the tumour environment. This hypothesis is also supported by the lack of effect on the other lineages (Fig. 7c, d, *IL13* data not shown). It has been demonstrated in various cancers that the tumour microenvironment expresses CXCL10 (IP-10), which leads to Th1-specific recruitment to the site mediated by the CXCL10 receptor CXCR3 expressed on Th1 cells. [31–33]. Thus, the changes in the proportion of Th1 cells specifically in different compartments may be based on their unique ability for tumour infiltration.

Our data indicates that NAC plays a role in activating and steering the immune system towards the tumour. It is tempting to speculate that patients with hypomethylation in the *IFNG* locus have better prognosis than those with hypermethylation. The short period of time from sample collection until present day prevents us from evaluating clinical parameters such as 5-year survival and relapse rates. However, histopathological response to NAC is a positive predictor of survival and, although a cause-and-effect relationship cannot be established, the data suggests that increased proportions of committed CD4^+^ T cells in the tumour regional lymph nodes after NAC translates into a better outcome. Further characterization of the immune response during NAC treatment and a longer follow-up is needed to fully comprehend the significance of this interaction.

## Conclusion

We found that patients with complete response to NAC treatment (CR) were hypomethylated in predictive CpG sites of CD4^+^ T cell signature loci. In addition, hypomethylation of signature effector CD4^+^ T cell loci were correlated with lower post-cystectomy tumour stage and overall better outcome, suggesting epigenetic staging of immune responses to be useful for clinical evaluation.

## Additional files

Additional file 1: **Table S1.** PCR assay-specific sequencing primers (DOCX 14 kb)

Additional file 2: **Figure S2.** PBMC comparison from the two time points TUR-B and RC. Comparing the two time points of intervention for all four loci. Methylation of CD4^+^ cells from PBMC obtained at TUR-B or Cystectomy were compared in a *IFNG* b *IL13* c *FOXP3* and d *IL17A*. Mann-Whitney test was used for statistical analysis. Bars show SEM. (TIF 1110 kb)

Additional file 3: **Figure S1.** Analysis of Cisplatin effect on healthy donors CD4^+^ T cells in vitro. CD4+ T cells were isolated from blood of healthy donors (*n* = 4) and cultured in vitro in the presence of neoadjuvant chemotherapy drug, Cisplatin. Stimulation at day 0 is indicated on *x*-axis. Sim = αCD3 and αCD28. Cisp 25 μM cisplatin. At day 6, all cultures were treated with αCD3 and αCD28, and cisplatin cultures (grey bars) received 25 μM cisplatin. The cells were harvested at day 12 for analysis. a Whole genome methylation was measured by 5mC ELISA. Corresponding cultures without cisplatin was used for normalization. Friedman test was used for statistical analysis. b Methylation of *IFNG* locus was measured. Unstimulated cells from Day 0 was used as normalization. (TIF 840 kb)

## Abbreviations

CD4: Cluster of differentiation 4
CR: Complete response
FOXP3: Forkhead box P3
IFNG: Interferon gamma
IL13: Interleukin 13
IL17A: Interleukin 17A
LN: Lymph node
MIBC: Muscle invasive bladder cancer
NAC: Neoadjuvant chemotherapy
PBMC: Peripheral blood mononuclear cells
RC: Radical cystectomy
Th: T helper cell
TIL: Tumour-infiltrating lymphocyte
Treg: T regulatory cell
TUR-B: Transurethral resection of the bladder
UBC: Urinary bladder cancer
