# Supplementary material for: Increased CD4+ T cell lineage commitment determined by CpG methylation correlates with better prognosis in urinary bladder cancer patients
Source: Clin Epigenetics. 2018 Aug 3;10:102. doi: 10.1186/s13148-018-0536-6 (PMC6076404; doi:10.1186/s13148-018-0536-6)
Supplement: Supplementary file 1 — Table S1. PCR assay-specific sequencing primers (DOCX 14 kb) [file 13148_2018_536_MOESM1_ESM.docx]

Table S1

| **Primer** | **Sequence 5’→3’** | **purpose** |
| --- | --- | --- |
| *IFNG* forward* | tat aaa aga aaa ggg ggg att tag | Locus specific PCR |
| *IFNG* reverse | caa cca aat tat ctc atc caa act | Locus specific PCR |
| *IFNG* sequencing | att atc tca tcc aaa ctt ta | Pyrosequencing primer |
| *IL13* forward | tgg ttt tgg gtg atg ttg att agt | Locus specific PCR |
| *IL13* reverse* | gga ttt att gag aag ggt tta ggg | Locus specific PCR |
| IL13 sequencing | gtt tgg gga ggt aaa gt | Pyrosequencing primer |
| *FOXP3* forward | tgg tga agt gga ttg ata gaa aag g | Locus specific PCR |
| *FOXP3* reverse* | aaa aaa aaa ccc aaa att tca a | Locus specific PCR |
| *FOXP3* sequencing | ggt ttg tgg gaa att g | Pyrosequencing primer |
| *IL17A* forward | gga tat ggt ttt tag gaa tat gaa | Locus specific PCR |
| *IL17A* reverse* | act cac cac caa taa aat ctt cc | Locus specific PCR |
| *IL17A* sequencing | ttt atg att tta ttg ggg | Pyrosequencing primer |

*= 5’ Biotinylated
