# Supplementary figures and images for: Increased CD4+ T cell lineage commitment determined by CpG methylation correlates with better prognosis in urinary bladder cancer patients
Source: Clin Epigenetics. 2018 Aug 3;10:102. doi: 10.1186/s13148-018-0536-6 (PMC6076404; doi:10.1186/s13148-018-0536-6)

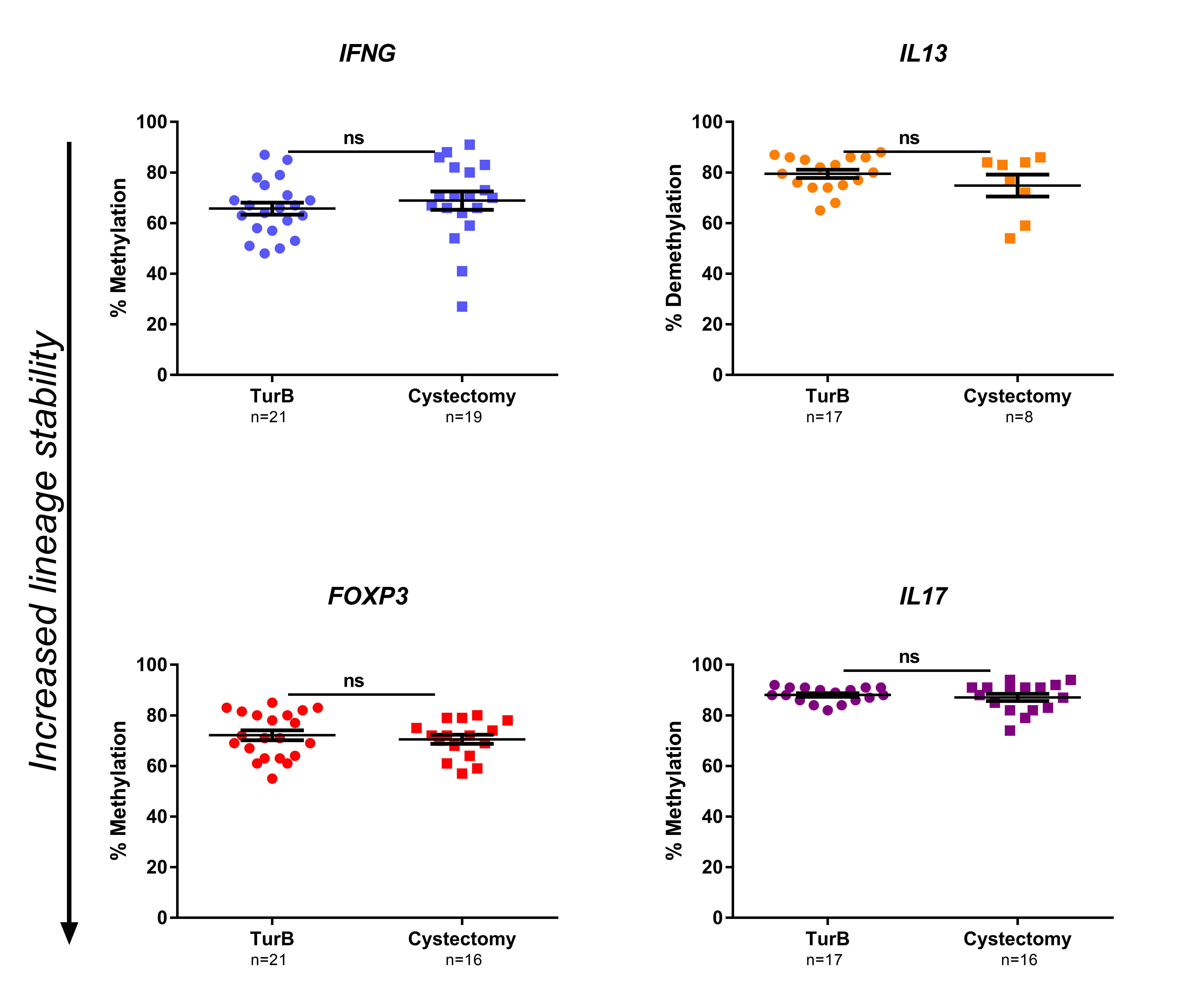

Supplement: Supplementary file 2 — Figure S2. PBMC comparison from the two time points TUR-B and RC. Comparing the two time points of intervention for all four loci. Methylation of CD4+ cells from PBMC obtained at TUR-B or Cystectomy were compared in a IFNG b IL13 c FOXP3 and d IL17A. Mann-Whitney test was used for statistical analysis. Bars show SEM. (TIF 1110 kb) [file 13148_2018_536_MOESM2_ESM.tif]

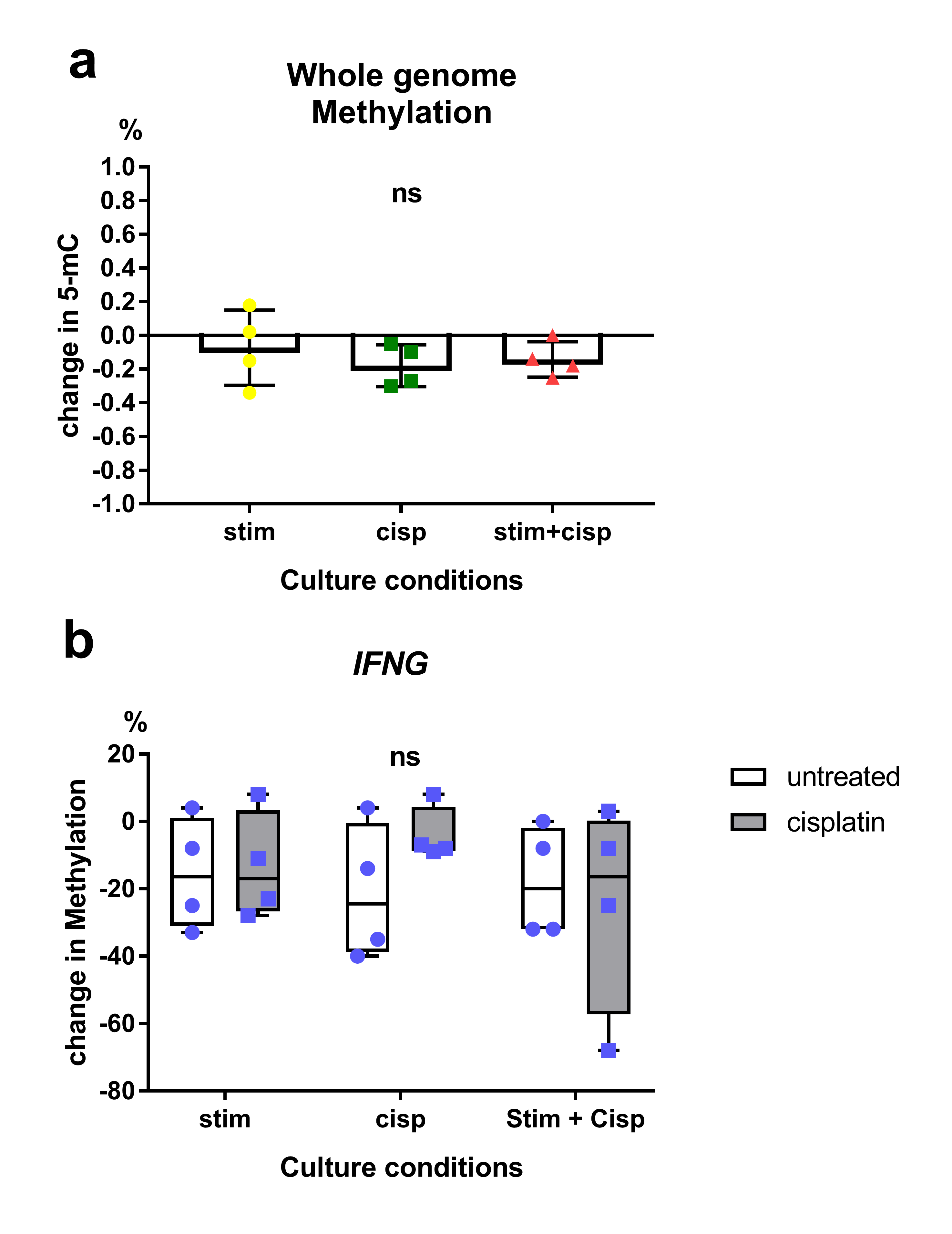

Supplement: Supplementary file 3 — Figure S1. Analysis of Cisplatin effect on healthy donors CD4+ T cells in vitro. CD4+ T cells were isolated from blood of healthy donors (n = 4) and cultured in vitro in the presence of neoadjuvant chemotherapy drug, Cisplatin. Stimulation at day 0 is indicated on x-axis. Sim = αCD3 and αCD28. Cisp 25 μM cisplatin. At day 6, all cultures were treated with αCD3 and αCD28, and cisplatin cultures (grey bars) received 25 μM cisplatin. The cells were harvested at day 12 for analysis. a Whole genome methylation was measured by 5mC ELISA. Corresponding cultures without cisplatin was used for normalization. Friedman test was used for statistical analysis. b Methylation of IFNG locus was measured. Unstimulated cells from Day 0 was used as normalization. (TIF 840 kb) [file 13148_2018_536_MOESM3_ESM.tif]
